# Supplementary material for: Transient measurement of phononic states with covariance-based stochastic spectroscopy
Source: Light Sci Appl. 2022 Mar 1;11:44. doi: 10.1038/s41377-022-00727-6 (PMC8885707; doi:10.1038/s41377-022-00727-6)
Supplement: Supplementary file 1 — Supplementary information [file 41377_2022_727_MOESM1_ESM.pdf]

Transient measurement of phononic states with covariance-based stochastic spectroscopy

Giorgia Sparapassi,[1,2] Stefano Cavaletto,[3] Jonathan Tollerud,[4,*] Angela Montanaro,[1,2] Filippo Glerean,[1,2] Alexandre Marciniak,[1,2] Francesca Giusti,[1,2] Shaul Mukamel,[3] and Daniele Fausti[1,2,†]

[1]*Physics Department, University of Trieste, 34127 Trieste, Italy*
[2]*Elettra-Sincrotrone Trieste S.C.p.A., 34149 Trieste, Italy*
[3]*Department of Chemistry and Department of Physics & Astronomy, University of California, Irvine, CA 92697, USA*
[4]*Optical Sciences Centre, Swinburne University, Melbourne, Australia, 3122*
(Dated: January 18, 2022)

S1. TIME- AND FREQUENCY-RESOLVED IMPULSIVE STIMULATED RAMAN SPECTROSCOPY SIGNAL

In this Supplementary Section, we derive the expressions for the impulsive stimulated Raman spectroscopy (ISRS) signal investigated in the main text. We derive expressions for a single phonon Ω and consider a manifold of vibrational states $|n\rangle$ of energy $\omega_n = \Omega(n+1/2)$. All expressions can be straightforwardly generalized by summing over all phonons.

In ISRS, two nonoverlapping pump and probe pulses, both off-resonant from electronic excited states, induce a sequence of impulsive stimulated Raman excitations, thereby coupling the vibrational states in the system. The effective interaction Hamiltonian in the rotating-wave approximation is given by [1]

$$\hat{H}_{\text{int}}(t) = -\hat{\alpha}\left[|\mathcal{E}_1(t+T)|^2 + |\mathcal{E}_2(t)|^2\right], \tag{S1}$$

with the effective polarizability operator $\hat{\alpha}$ and classical electric fields

$$\mathcal{E}_i(t) = E_i(t)\,\mathrm{e}^{-\mathrm{i}\omega_{i0}t}, \tag{S2}$$

$i \in \{1, 2\}$, of envelopes $E_i(t)$, carrier frequencies ω_{i0}, and associated Fourier transforms

$$\tilde{\mathcal{E}}_i(\omega) = \int \mathrm{d}t\,\mathcal{E}_i(t)\,\mathrm{e}^{\mathrm{i}\omega t}, \qquad \mathcal{E}_i(t) = \frac{1}{2\pi}\int \mathrm{d}\omega\,\tilde{\mathcal{E}}_i(\omega)\,\mathrm{e}^{-\mathrm{i}\omega t}. \tag{S3}$$

The pump $\mathcal{E}_1(t+T)$ and probe $\mathcal{E}_2(t)$ pulses are centered around $t_{10} = -T$ and $t_{20} = 0$, respectively.

The ladder diagrams and level scheme of the ISRS signal are depicted in Fig. S1. For nonoverlapping pulses, the system is first excited by the pump pulse $\mathcal{E}_1(t+T)$ from an initial equilibrium state $\hat{\rho}^{(0)}$ to a coherent superposition of vibrational states. To first order in perturbation theory, the interaction with the pump pulse and the resulting evolution of the system's density matrix $\hat{\rho}(t)$, of elements $\rho_{nn'}(t)$, are given by

$$\rho_{nn'}(t) = \rho_{0,nn'} + \mathrm{i}\int_{-\infty}^{t} \mathrm{d}t'\,\mathrm{e}^{-\mathrm{i}\omega_{nn'}(t-t')}\,\mathrm{e}^{-\gamma(t-t')}\,\alpha_{nn'}\,|\mathcal{E}_1(t'+T)|^2\left(\rho^{(0)}_{n'n'} - \rho^{(0)}_{nn}\right), \tag{S4}$$

with the transition energies $\omega_{nn'} = \Omega(n-n')$ and decay rates γ. The two terms in the above sum, $\rho^{(0)}_{n'n'}$ and $\rho^{(0)}_{nn}$, are associated with the top and bottom diagrams in Fig. S1, respectively. For nonoverlapping pulses, we can extend the above integral to $t \to +\infty$, so that Eq. (S4) can be recast as

$$\rho_{nn'}(t) = \rho_{0,nn'} + \mathrm{i}\,\mathrm{e}^{-\mathrm{i}\omega_{nn'}(t+T)}\,\mathrm{e}^{-\gamma(t+T)}\int_{-\infty}^{+\infty} \mathrm{d}t'\,\mathrm{e}^{\mathrm{i}\omega_{nn'}t'}\,\mathrm{e}^{\gamma t'}\,\alpha_{nn'}\,|\mathcal{E}_1(t')|^2\left(\rho^{(0)}_{n'n'} - \rho^{(0)}_{nn}\right). \tag{S5}$$

The pump pulse used in the experiment is a coherent pulse of Gaussian envelope

$$\mathcal{E}_1(t) = E_{10}\,\mathrm{e}^{-\sigma_1^2 t^2/2}\,\mathrm{e}^{-\mathrm{i}\omega_{10}t}, \tag{S6}$$

* Email: jtollerud@swin.edu.au
† Email: daniele.fausti@elettra.eu

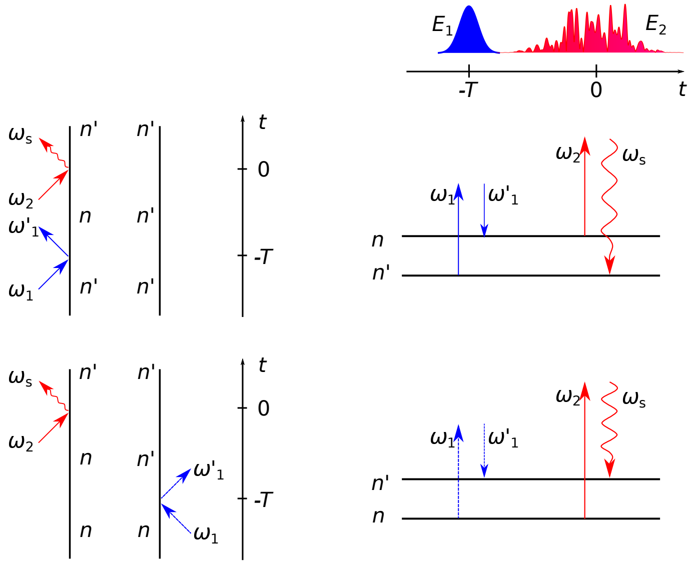

Figure S1. Ladder diagrams and level schemes for the third-order ISRS signal.

with central frequency $\omega_{10} = 230\,\mathrm{THz}$ (1300 nm), peak strength E_{10}, and bandwidth $\sigma_1 = 2.5\,\mathrm{THz}$ (110 fs duration). For such coherent Gaussian pulse, and for a system in the initial thermal state

$$\hat{\rho}^{(0)} = \sum_n \rho^{(0)}_{nn}\,|n\rangle\langle n| = \sum_n \frac{\mathrm{e}^{-\beta\Omega n}}{1-\mathrm{e}^{-\beta\Omega n}}\,|n\rangle\langle n|, \tag{S7}$$

with initial populations $\rho^{(0)}_{nn}$, $\beta = 1/(k_\mathrm{B}T_\mathrm{e})$, Boltzmann constant k_B, and temperature T_e, Eq. (S5) reduces to

$$\rho_{nn'}(t) = \rho^{(0)}_{nn}\,\delta_{nn'} + \rho^{(1)}_{nn'}\,\mathrm{e}^{-\mathrm{i}\Omega(n-n')(t+T)}\,\mathrm{e}^{-\gamma(t+T)} \tag{S8}$$

in terms of the first-order perturbation

$$\rho^{(1)}_{nn'} = \mathrm{i}\,|E_{10}|^2\,\frac{\sqrt{\pi}}{\sigma_1}\,\mathrm{e}^{-\Omega^2(n-n')^2/(4\sigma_1^2)}\,\alpha_{nn'}\,\left(\rho^{(0)}_{n'n'} - \rho^{(0)}_{nn}\right). \tag{S9}$$

Due to the Gaussian envelope $\mathrm{e}^{-\Omega^2(n-n')^2/(4\sigma_1^2)}$ in Eq. (S9) and its finite bandwidth σ_1, we focus only on the matrix elements $\rho_{nn'}(t)$ for which either $n' = n$ or $n' = n \pm 1$, and neglect all remaining off-diagonal elements in Eq. (S8).

The evolution of the superposition of vibrational states generated by the pump pulse is monitored by a subsequent stimulated Raman process induced by the probe pulse $\mathcal{E}_2(t)$. The frequency-dispersed heterodyne-detected signal is defined as the time-integrated rate of change of photon number in the probe pulse [2]

$$S(\omega_\mathrm{s}, T) = -\int \mathrm{d}t\,\left\langle \frac{\mathrm{d}\hat{N}^{(2)}_{\omega_\mathrm{s}}(t)}{\mathrm{d}t} \right\rangle, \tag{S10}$$

where $\hat{N}_\omega = \hat{a}^\dagger_\omega \hat{a}_\omega$ denotes the number operator for a photon mode with frequency ω. The superscript "(2)" in $\hat{N}^{(2)}_{\omega_\mathrm{s}}$ stresses that the modes are restricted to those occupied by the probe pulse $\mathcal{E}_2(t)$. By using the Heisenberg equations of motion for the photon number operator, the signal at the detected frequency ω_s reads

$$S(\omega_\mathrm{s}) = 2\,\mathbf{Im}\left\{\tilde{\mathcal{E}}^*_2(\omega_\mathrm{s}) \int \mathrm{d}t\,\mathcal{E}_2(t)\mathrm{e}^{\mathrm{i}\omega_\mathrm{s}t}\langle\hat{\alpha}(t)\rangle\right\}, \tag{S11}$$

with the time-dependent polarizability

$$\langle\hat{\alpha}(t)\rangle = \mathrm{Tr}\{\hat{\alpha}\,\hat{\rho}(t)\} = \sum_{nn'} \alpha_{n'n}\,\rho_{nn'}(t). \tag{S12}$$

For nonoverlapping pulses, by expressing the elements of the density matrix via Eq. (S8), the time- and frequency-dependent ISRS signal can be recast as

$$\begin{aligned} S_{\mathrm{ISRS}}(\omega_{\mathrm{s}},T) = {} & 2\,\mathbf{Im}\left\{\tilde{\mathcal{E}}_2^*(\omega_{\mathrm{s}})\,\tilde{\mathcal{E}}_2(\omega_{\mathrm{s}}-\Omega)\,A\,\mathrm{e}^{-\mathrm{i}\Omega T}\,\mathrm{e}^{-\gamma T}\right\} \\ & + 2\,\mathbf{Im}\left\{\tilde{\mathcal{E}}_2^*(\omega_{\mathrm{s}})\,\tilde{\mathcal{E}}_2(\omega_{\mathrm{s}}+\Omega)\,A^*\,\mathrm{e}^{\mathrm{i}\Omega T}\,\mathrm{e}^{-\gamma T}\right\}, \end{aligned} \tag{S13}$$

where

$$A = \sum_n \alpha_{n,n+1}\,\rho^{(1)}_{n+1,n} = \mathrm{i}\,|E_{10}|^2\,\frac{\sqrt{\pi}}{\sigma_1}\,\mathrm{e}^{-\Omega^2/(4\sigma_1^2)}\sum_n |\alpha_{n,n+1}|^2\left(\rho^{(0)}_{nn}-\rho^{(0)}_{n+1,n+1}\right) \tag{S14}$$

contains the amplitude and phase information of the vibrational coherence generated by the pump pulse. For $\alpha_{n,n+1} \approx \tilde{\alpha}$, this coherence term A reduces to

$$A \approx \mathrm{i}\,|E_{10}|^2\,\frac{\sqrt{\pi}}{\sigma_1}\,\mathrm{e}^{-\Omega^2/(4\sigma_1^2)}\,|\tilde{\alpha}|^2\,\frac{1}{1-\mathrm{e}^{-\beta\Omega}}. \tag{S15}$$

Additional features appear in the presence of overlapping pump and probe pulses, which are responsible for the hyperbola branches in the (ω_{s}, T) plane observed in the experiment. These features have been previously analyzed [3] and are not the focus of this work.

S2. MODELING OF THE STOCHASTIC PROBE PULSE

The stochastic probe pulse used in the experiment is obtained from an initial coherent pulse, here modeled as a Gaussian pulse

$$\tilde{g}_2(\omega) = \mathrm{e}^{-\omega^2/(2\sigma_2^2)} \tag{S16}$$

of bandwidth $\sigma_2 = 6.6\,\mathrm{THz}$ (40 fs duration). The pulse spectral phase $\varphi(\omega)$ is shaped by a pulse shaper with finite resolution $R = 0.1\,\mathrm{THz}$, leading to the shaped stochastic pulse

$$\tilde{\mathcal{E}}_{2,\mathrm{sh}}(\omega) = \tilde{E}_{20}\,\tilde{g}_2(\omega-\omega_{20})\,\mathrm{e}^{\mathrm{i}\varphi(\omega)}, \tag{S17}$$

with central frequency $\omega_{20} = 375\,\mathrm{THz}$ (800 nm) and peak strength E_{20}. In Sec. S5, a pulse with noise present only on part of its spectral width will be considered.

The spectral phase $\varphi(\omega)$ is obtained by generating an array of N independent random numbers φ_k for a discrete set of frequencies $\omega_k = \omega_{20} + kR$, $k \in \{-N/2, -N/2+1, \cdots, N/2-1\}$. Each number is randomly distributed in the interval $[-\bar{\varphi}, \bar{\varphi}]$, with vanishing expectation value $\langle\varphi_k\rangle = 0$ and with $\langle\varphi_k\,\varphi_{k'}\rangle = \delta_{kk'}\,\bar{\varphi}^2/3$. This is convolved with a Gaussian smoothing function with width R, resulting in

$$\varphi(\omega) = \sum_k \varphi_k\,\mathrm{e}^{-(\omega-\omega_k)^2/(2R^2)}. \tag{S18}$$

The expectation value of the spectral phase is thus equal to

$$\langle\varphi(\omega)\rangle = 0 \tag{S19}$$

and its correlation function is given by

$$\langle\varphi(\omega)\varphi(\omega')\rangle = \langle\varphi^2\rangle\,\mathrm{e}^{-(\omega-\omega')^2/(4R^2)}, \tag{S20}$$

with the variance

$$\langle\varphi^2\rangle = \frac{\bar{\varphi}^2}{3}\sum_k \mathrm{e}^{-k^2}. \tag{S21}$$

In order to interpret the spectra obtained by the covariance approach used in the main text, we introduce the two- and four-point correlation functions of $\mathrm{e}^{\mathrm{i}\varphi(\omega)}$, defined as

$$\tilde{F}_2(\omega_1,\omega_2) \doteq \langle\mathrm{e}^{-\mathrm{i}\varphi(\omega_1)}\,\mathrm{e}^{\mathrm{i}\varphi(\omega_2)}\rangle \tag{S22}$$

and

$$\tilde{F}_4(\omega_1,\omega_2,\omega_3,\omega_4) \doteq \langle \mathrm{e}^{-\mathrm{i}\varphi(\omega_1)}\,\mathrm{e}^{\mathrm{i}\varphi(\omega_2)}\,\mathrm{e}^{-\mathrm{i}\varphi(\omega_3)}\,\mathrm{e}^{\mathrm{i}\varphi(\omega_4)}\rangle, \tag{S23}$$

respectively. To calculate these field correlation functions, we first introduce the correlation function $\langle\dot{\varphi}(\omega)\dot{\varphi}(\omega')\rangle$ of the first derivative of the spectral phase $\dot{\varphi}(\omega) = \mathrm{d}\varphi/\,\mathrm{d}\omega$. The stochastic probe pulse employed here is a wide-sense stationary process, for which

$$\langle\dot{\varphi}(\omega)\dot{\varphi}(\omega')\rangle = \tilde{q}(\omega-\omega') = \frac{\langle\varphi^2\rangle}{2R^2}\,\mathrm{e}^{-(\omega-\omega')^2/(4R^2)}\left(1-\frac{(\omega-\omega')^2}{2R^2}\right) \tag{S24}$$

only depends on the difference $\omega-\omega'$. We thus introduce the function

$$\tilde{p}(\omega) \doteq \int_0^\omega \mathrm{d}x \int_0^\omega \mathrm{d}y\,\langle\dot{\varphi}(x)\dot{\varphi}(y)\rangle = 2\,\langle\varphi^2\rangle\left(1-\mathrm{e}^{-\omega^2/(4R^2)}\right) \tag{S25}$$

and use the second-order cumulant expansion to write the two- [Eq. (S22)] and four-point [Eq. (S23)] correlation functions in terms of $\tilde{p}(\omega)$ [4], leading to the following identities:

$$\tilde{F}_2(\omega_1,\omega_2) = \mathrm{e}^{-\tilde{p}(\omega_1-\omega_2)/2} \tag{S26}$$

and

$$\tilde{F}_4(\omega_1,\omega_2,\omega_3,\omega_4) = \mathrm{e}^{-[\tilde{p}(\omega_1-\omega_2)-\tilde{p}(\omega_1-\omega_3)+\tilde{p}(\omega_1-\omega_4)+\tilde{p}(\omega_2-\omega_3)-\tilde{p}(\omega_2-\omega_4)+\tilde{p}(\omega_3-\omega_4)]/2}. \tag{S27}$$

We notice that $\tilde{F}_2(\omega_1,\omega_2) = \tilde{F}_2(\omega_1-\tilde{\omega},\omega_2-\tilde{\omega})$ and $\tilde{F}_4(\omega_1,\omega_2,\omega_3,\omega_4) = \tilde{F}_4(\omega_1-\tilde{\omega},\omega_2-\tilde{\omega},\omega_3-\tilde{\omega},\omega_4-\tilde{\omega})$ for any $\tilde{\omega}$.

The two-point correlation function $F_{2,\mathrm{sh}}(\omega,\omega')$ of the spectral pulse $\tilde{\mathcal{E}}_{2,\mathrm{sh}}(\omega)$ can be calculated in terms of $\tilde{F}_2(\omega,\omega')$ and is equal to

$$\begin{aligned} F_{2,\mathrm{sh}}(\omega,\omega') &\doteq \langle\tilde{\mathcal{E}}^*_{2,\mathrm{sh}}(\omega)\tilde{\mathcal{E}}_{2,\mathrm{sh}}(\omega')\rangle = |\tilde{E}_{20}|^2\,\tilde{g}_2(\omega-\omega_{20})\,\tilde{g}_2(\omega'-\omega_{20})\,\tilde{F}_2(\omega,\omega') \\ &= |\tilde{E}_{20}|^2\,\tilde{g}_2(\omega-\omega_{20})\,\tilde{g}_2(\omega'-\omega_{20})\,\exp\left(-\langle\varphi^2\rangle\left(1-\mathrm{e}^{-(\omega-\omega')^2/(4R^2)}\right)\right). \end{aligned} \tag{S28}$$

Figures S2 and S3 show the frequency and time properties of a numerical simulation of the stochastic pulses in Eq. (S17), for values of the variance $\langle\varphi^2\rangle = (\pi/4)^2$ and $\langle\varphi^2\rangle = (\pi/2)^2$, respectively. We notice in particular that, due to the method used to generate the stochastic spectral phases, these do not uniformly cover the whole phase interval $[-\pi,\,\pi]$. This is evident when $\langle\varphi^2\rangle = (\pi/4)^2$, and is still the case even for $\langle\varphi^2\rangle = (\pi/2)^2$. As a result, the amplitude of the average stochastic pulse $\langle\tilde{\mathcal{E}}_{2,\mathrm{sh}}(\omega)\rangle$ does not vanish, even though its peak value reduces for increasing values of $\langle\varphi^2\rangle$. In time domain, this results in the appearance of a central peak surrounded by a noisy tail: this central peak survives in the envelope of the average stochastic pulse $\langle E_{2,\mathrm{sh}}(t)\rangle$, whereas the noisy tails average out to 0.

The two panels in Fig. S4 show the associated correlation functions $\langle\dot{\varphi}(\omega)\dot{\varphi}(\omega')\rangle$ [Eq. (S24)] and $\langle\tilde{\mathcal{E}}^*_{2,\mathrm{sh}}(\omega)\tilde{\mathcal{E}}_{2,\mathrm{sh}}(\omega')\rangle$ [Eq. (S28)]. The curves display the behaviour of the correlation functions for $\langle\varphi^2\rangle = (\pi/4)^2$ and $\langle\varphi^2\rangle = (\pi/2)^2$. The correlation function of the first derivative of the spectral phase shows an oscillatory behavior, with a strong positive central peak surrounded by two negative peaks. For very close frequencies ω and ω' lying within the width of the central peak, the associated spectral phases will have correlated first derivatives $\dot{\varphi}(\omega)$ and $\dot{\varphi}(\omega')$ with the same sign. However, for increasing frequency differences, the spectral phases do not become directly uncorrelated: when the frequencies ω and ω' lie within the width of the second negative peak, this means that the associated spectral phases are still correlated, with first derivatives $\dot{\varphi}(\omega)$ and $\dot{\varphi}(\omega')$ exhibiting opposite sign. As a result, the autocorrelation of the field $\langle\tilde{\mathcal{E}}^*_{2,\mathrm{sh}}(\omega)\tilde{\mathcal{E}}_{2,\mathrm{sh}}(\omega')\rangle$ features one strong central peak. Out of this peak, however, the stochastic pulse still presents a nonvanishing autocorrelation function. The amplitude of this residual autocorrelation decreases with increasing values of the variance $\langle\varphi^2\rangle$.

In the experiment, the stochastic probe pulse was obtained by shaping its spectral phase. Nevertheless, the measured spectral intensity $|\tilde{\mathcal{E}}_2(\omega)|^2$ features chaotic oscillations as apparent in Fig. 1b in the main text. This is due to the finite resolution Q of the optical detector. The detected field is given by the convolution of the stochastic probe pulse $\tilde{\mathcal{E}}_{2,\mathrm{sh}}(\omega)$ generated by the pulse shaper and the function $q_Q(\omega)$ modeling the finite resolution of the optical detector

$$\tilde{\mathcal{E}}_2(\omega) = \int \mathrm{d}\omega'\,\tilde{\mathcal{E}}_{2,\mathrm{sh}}(\omega-\omega')\,q_Q(\omega'). \tag{S29}$$

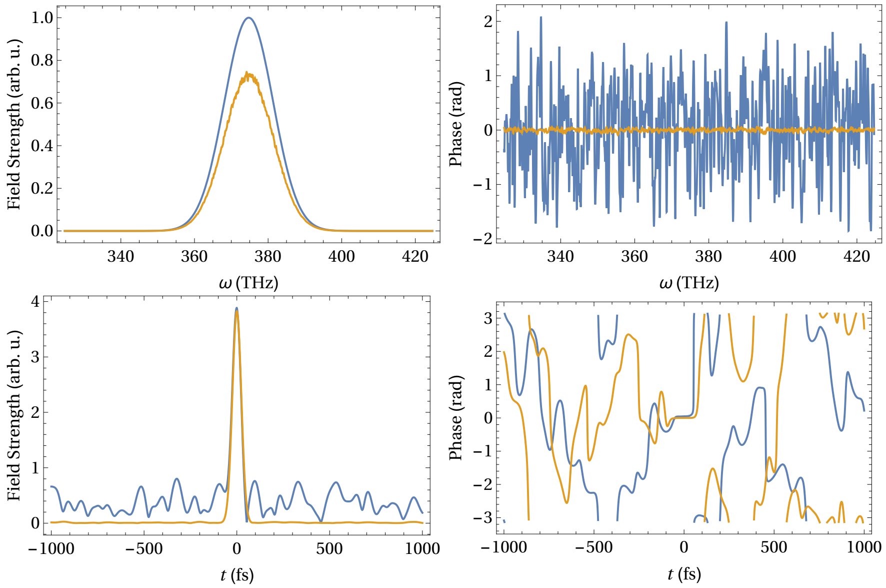

Figure S2. Numerical simulation of the stochastic probe pulses in Eq. (S17) for $\langle\varphi^2\rangle = (\pi/4)^2$. The first row shows the spectral properties of the pulse, by exhibiting (left) the amplitude and (right) the phase of (blue) one realization of the stochastic probe pulse $\tilde{\mathcal{E}}_{2,\mathrm{sh}}(\omega)$ and (yellow) an average over 1,000 independent realizations. The second row shows the temporal properties of the same stochastic pulses, i.e., (left) the amplitude and (right) the phase of (blue) one realization of the envelope $E_{2,\mathrm{sh}}(t)$ of the stochastic probe pulse and (yellow) an average over 1,000 independent realizations.

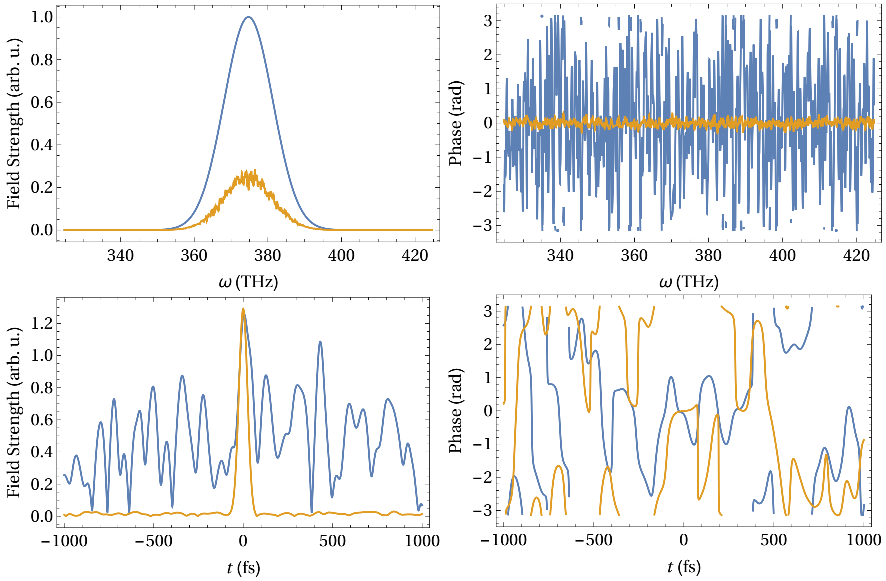

Figure S3. Numerical simulation of the stochastic probe pulses in Eq. (S17) for $\langle\varphi^2\rangle = (\pi/2)^2$. The different panels show the same properties of the pulses as in Fig. S2.

The spectral gating function $q_Q(\omega)$ acts over the fast random oscillations of $\mathrm{e}^{\mathrm{i}\varphi}$, affecting both amplitude and phase of $\tilde{\mathcal{E}}_2(\omega)$ and leading the the intensity fluctuations depicted in Fig. 1b in the main text. The two- and four-point correlation functions of the detected field are thus given by

$$\begin{aligned} F_2(\omega_1,\omega_2) &\doteq \langle \tilde{\mathcal{E}}_2^*(\omega_1)\tilde{\mathcal{E}}_2(\omega_2)\rangle \\ &= |\tilde{E}_{20}|^2\, \tilde{g}_2(\omega_1-\omega_{20})\, \tilde{g}_2(\omega_2-\omega_{20}) \int \mathrm{d}\omega_1' \int \mathrm{d}\omega_2'\, \tilde{F}_2(\omega_1-\omega_1',\omega_2-\omega_2')\, q_Q(\omega_1')\, q_Q(\omega_2') \end{aligned} \tag{S30}$$

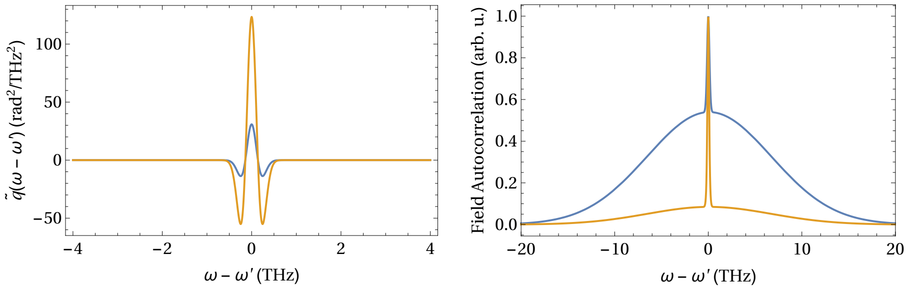

Figure S4. Correlation functions (left) $\tilde{q}(\omega-\omega') = \langle\dot{\varphi}(\omega)\dot{\varphi}(\omega')\rangle$ [Eq. (S24)] and (right) $F_{2,\mathrm{sh}}(\omega,\omega') = \langle\tilde{\mathcal{E}}^*_{2,\mathrm{sh}}(\omega)\tilde{\mathcal{E}}_{2,\mathrm{sh}}(\omega')\rangle$ [Eq. (S28)] of the stochastic probe pulses in Eq. (S17) for (blue) $\langle\varphi^2\rangle = (\pi/4)^2$ and (yellow) $\langle\varphi^2\rangle = (\pi/2)^2$.

and

$$\begin{aligned} &F_4(\omega_1,\omega_2,\omega_3,\omega_4) \doteq \langle\tilde{\mathcal{E}}^*_2(\omega_1)\tilde{\mathcal{E}}_2(\omega_2)\tilde{\mathcal{E}}^*_2(\omega_3)\tilde{\mathcal{E}}_2(\omega_4)\rangle \\ &= |\tilde{E}_{20}|^4\,\tilde{g}_2(\omega_1-\omega_{20})\,\tilde{g}_2(\omega_2-\omega_{20})\,\tilde{g}_2(\omega_3-\omega_{20})\,\tilde{g}_2(\omega_4-\omega_{20}) \\ &\quad\times\int \mathrm{d}\omega'_1\int \mathrm{d}\omega'_2\int \mathrm{d}\omega'_3\int \mathrm{d}\omega'_4\,\tilde{F}_4(\omega_1-\omega'_1,\omega_2-\omega'_2,\omega_3-\omega'_3,\omega_4-\omega'_4)\,q_Q(\omega'_1)\,q_Q(\omega'_2)\,q_Q(\omega'_3)\,q_Q(\omega'_4), \end{aligned} \tag{S31}$$

where we moved the broadband Gaussian envelopes $\tilde{g}(\omega)$ out of the integrals. In the following, we will model

$$q_Q(\omega) = \frac{1}{Q}\,\mathrm{rect}\left(\frac{\omega}{Q}\right) \tag{S32}$$

as a rectangular function of width $Q = 0.1\,\mathrm{THz}$ and unit area, and we will use Eqs. (S26) and (S27) to perform the integrals in Eqs. (S30) and (S31) numerically.

Equation (S29) has a similar structure as models recently employed to describe the stochastic features of hard-x-ray free-electron-laser pulses generated by the self-amplified spontaneous emission methods [4–7]. In these x-ray models, a spectral gating function is included in order to reproduce the finite pulse duration and the corresponding coherence width of the spikes in the frequency envelope of the pulse. This is achieved by setting a spectral correlation length (the equivalent of Q) much larger than the correlation length of the pure phase fluctuations (the equivalent of R). This differs from this work, where the spectral gating function is included to reproduce the finite resolution of the optical detector, and where we set $Q = R$. This determines the shape of the two- and four-point field correlation functions, which will be discussed in the following.

S3. COVARIANCE-BASED SPECTROSCOPY AND PEARSON COEFFICIENTS

In this Supplementary Section, we define the Pearson coefficients analyzed in the main text. The experiment is repeated for independent realizations of a stochastic probe, and the spectra are quantified via the Pearson coefficient

$$P(I_1(\omega_{\mathrm{s}1}), I_2(\omega_{\mathrm{s}2})) = \frac{\langle I_1(\omega_{\mathrm{s}1})I_2(\omega_{\mathrm{s}2})\rangle - \langle I_1(\omega_{\mathrm{s}1})\rangle\langle I_2(\omega_{\mathrm{s}2})\rangle}{\sigma_1(\omega_{\mathrm{s}1})\sigma_2(\omega_{\mathrm{s}2})}, \tag{S33}$$

where $I_1(\omega_{\mathrm{s}1})$ and $I_2(\omega_{\mathrm{s}2})$ can either be the detected input intensity of the probe pulse [$\tilde{\mathcal{E}}_2(\omega_{\mathrm{s}})$ in Eq. (S29)] before interaction with the sample

$$I_{\mathrm{in}}(\omega_{\mathrm{s}}) = \tilde{\mathcal{E}}^*_2(\omega_{\mathrm{s}})\tilde{\mathcal{E}}_2(\omega_{\mathrm{s}}), \tag{S34}$$

or the detected output intensity of the probe pulse after transmitting through the sample

$$I_{\mathrm{out}}(\omega_{\mathrm{s}}, T) = I_{\mathrm{in}}(\omega_{\mathrm{s}}) + S(\omega_{\mathrm{s}}, T). \tag{S35}$$

In the definition of the Pearson coefficient, $\sigma_i(\omega_{\mathrm{s}i})$ denotes the standard deviation of $I_i(\omega_i)$ given by

$$\sigma_i(\omega_{\mathrm{s}i}) = \sqrt{\langle I_i^2(\omega_{\mathrm{s}i})\rangle - \langle I_i(\omega_{\mathrm{s}i})\rangle^2}. \tag{S36}$$

In the following, we will focus on the autocorrelation of the input intensity,

$$P(I_{\text{in}}(\omega_{\text{s1}}), I_{\text{in}}(\omega_{\text{s2}})) = \frac{\langle I_{\text{in}}(\omega_{\text{s1}}) I_{\text{in}}(\omega_{\text{s2}})\rangle - \langle I_{\text{in}}(\omega_{\text{s1}})\rangle \langle I_{\text{in}}(\omega_{\text{s2}})\rangle}{\sigma_{\text{in}}(\omega_{\text{s1}})\sigma_{\text{in}}(\omega_{\text{s2}})}, \tag{S37}$$

and the cross-correlation

$$\begin{aligned} P(I_{\text{in}}(\omega_{\text{s1}}, T), I_{\text{out}}(\omega_{\text{s2}}, T)) = & \frac{\langle I_{\text{in}}(\omega_{\text{s1}}) I_{\text{in}}(\omega_{\text{s2}})\rangle - \langle I_{\text{in}}(\omega_{\text{s1}})\rangle \langle I_{\text{in}}(\omega_{\text{s2}})\rangle}{\sigma_{\text{in}}(\omega_{\text{s1}})\sigma_{\text{out}}(\omega_{\text{s2}}, T)} \\ & + \frac{\langle I_{\text{in}}(\omega_{\text{s1}}) S(\omega_{\text{s2}}, T)\rangle - \langle I_{\text{in}}(\omega_{\text{s1}})\rangle \langle S(\omega_{\text{s2}}, T)\rangle}{\sigma_{\text{in}}(\omega_{\text{s1}})\sigma_{\text{out}}(\omega_{\text{s2}}, T)} \end{aligned} \tag{S38}$$

of input and output intensities. In all above formulas, the signal–signal correlation terms have not been included since they are weaker than the correlation between input intensity and signal.

The cross-correlation $P(I_{\text{in}}(\omega_{\text{s1}}, T), I_{\text{out}}(\omega_{\text{s2}}, T))$ corresponds to three-dimensional matrix $\rho_{\text{c}}(\omega_{\text{IN}}, \omega_{\text{OUT}}, T)$ displayed in Fig. 1c in the main text, where $\omega_{\text{s1}} = \omega_{\text{IN}}$ and $\omega_{\text{s2}} = \omega_{\text{OUT}}$. As shown in Eq. (S38), this cross-correlation contains two main contributions, owing to the autocorrelation of the input pulse and the cross-correlation between input intensity and ISRS signal, respectively. This latter term,

$$P_{I,S}(\omega_{\text{s1}}, \omega_{\text{s2}}, T) = \frac{\langle I_{\text{in}}(\omega_{\text{s1}}) S_{\text{ISRS}}(\omega_{\text{s2}}, T)\rangle - \langle I_{\text{in}}(\omega_{\text{s1}})\rangle \langle S_{\text{ISRS}}(\omega_{\text{s2}}, T)\rangle}{\sigma_{\text{in}}(\omega_{\text{s1}})\, \sigma_{\text{out}}(\omega_{\text{s2}})}, \tag{S39}$$

is responsible for the off-diagonal lines appearing in the bottom-right quadrant of the correlation map displayed in Fig. 1c in the main text, and is therefore the term we will focus on in the following.

S4. AUTO- AND CROSS-CORRELATIONS FOR A STOCHASTIC PROBE WITH NOISE DISTRIBUTED IN THE ENTIRE SPECTRAL BANDWIDTH

In the main text, we presented results for the partially stochastic pulses of Fig. 1b, where the pulse is shaped only at frequencies larger than 370 THz. For modeling purposes, we here start by describing the case of a pulse with noise distributed in its entire spectral bandwidth. The case of a partially shaped pulse is presented in Sec. S5.

In this Supplementary Section, we therefore consider the stochastic probe pulse of Eq. (S17), with noise distributed on the whole frequency spectrum. We set $\langle \varphi^2 \rangle = (\pi/2)^2$ and include the effect of the finite resolution of the optical detector via Eq. (S29) as a rectangular function $q_Q(\omega)$ [Eq. (S32)] of width $Q = 0.1$ THz. Different choices of the function modeling the optical detector do not modify our conclusions. We consider nonoverlapping pump and probe pulses, so that the signal $S(\omega_{\text{s}}, T)$ contributing to the output intensity $I_{\text{out}}(\omega_{\text{s}}, T)$ is given by $S_{\text{ISRS}}(\omega_{\text{s}}, T)$ in Eq. (S13). The Pearson coefficients for auto- and cross-correlation of input and output intensities are exemplified for a system with a single phonon of frequency $\Omega = 10$ THz and lifetime $1/\gamma = 1.6$ ps.

A simulation of the autocorrelation of the detected input intensity, given by the Pearson coefficient $P(I_{\text{in}}(\omega_{\text{s1}}), I_{\text{in}}(\omega_{\text{s2}}))$ in Eq. (S37), is shown in Fig. S5. This is calculated in terms of the two- [Eq. (S30)] and

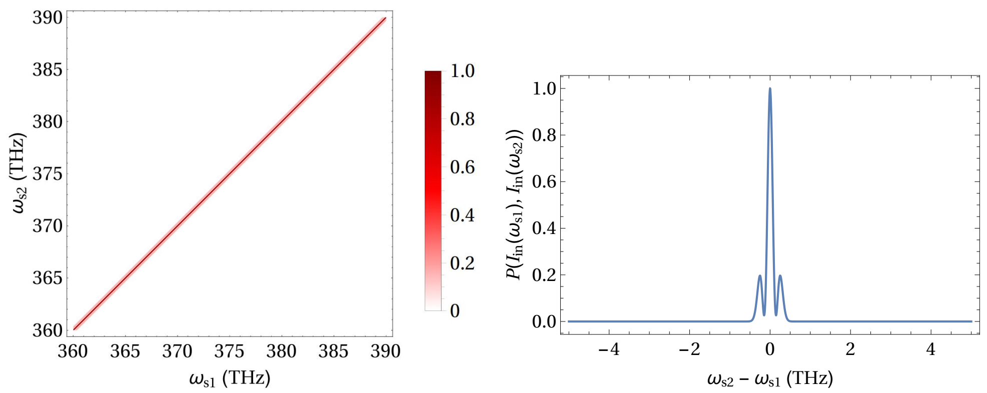

Figure S5. Pearson coefficient $P(I_{\text{in}}(\omega_{\text{s1}}), I_{\text{in}}(\omega_{\text{s2}}))$ of the autocorrelation of the detected input intensity for a fully modulated stochastic probe pulse (left) as a function of the two frequencies ω_{s1} and ω_{s2} and (right) as a function of the frequency difference $\omega_{\text{s1}} - \omega_{\text{s2}}$ for $(\omega_{\text{s1}} + \omega_{\text{s2}})/2 = 374$ THz.

four-point [Eq. (S31)] correlation functions of the detected field as

$$P(I_{\rm in}(\omega_{\rm s1}), I_{\rm in}(\omega_{\rm s2})) = \frac{F_4(\omega_{\rm s1},\omega_{\rm s1},\omega_{\rm s2},\omega_{\rm s2}) - F_2(\omega_{\rm s1},\omega_{\rm s1})\,F_2(\omega_{\rm s2},\omega_{\rm s2})}{\sigma_{\rm in}(\omega_{\rm s1})\,\sigma_{\rm in}(\omega_{\rm s2})}, \tag{S40}$$

with the standard deviation

$$\sigma_{\rm in}(\omega_{{\rm s}i}) = \sqrt{F_4(\omega_{{\rm s}i},\omega_{{\rm s}i},\omega_{{\rm s}i},\omega_{{\rm s}i}) - [F_2(\omega_{{\rm s}i},\omega_{{\rm s}i})]^2}. \tag{S41}$$

Figure S5 exhibits a strong peak along the main diagonal $\omega_{\rm s1} = \omega_{\rm s2}$ with width determined by the pulse-shaper and optical-detector resolutions. We notice also the presence of additional, lower-intensity peaks around the central one, as visible on the right panel of Fig. S5.

When input and output intensity are cross-correlated, the resulting signal carries the additional contribution due to $P_{I,S}(\omega_{\rm s1},\omega_{\rm s2},T)$ from Eq. (S39). With the expression for the signal in Eq. (S13), this contribution reads

$$P_{I,S}(\omega_{\rm s1},\omega_{\rm s2},T) = \frac{2\,\mathbf{Im}\left\{G(\omega_{\rm s1},\omega_{\rm s2},\varOmega)\,A\,{\rm e}^{-{\rm i}\varOmega T}\,{\rm e}^{-\gamma T} + G(\omega_{\rm s1},\omega_{\rm s2},-\varOmega)\,A^*\,{\rm e}^{{\rm i}\varOmega T}\,{\rm e}^{-\gamma T}\right\}}{\sigma_{\rm in}(\omega_{\rm s1})\,\sigma_{\rm out}(\omega_{\rm s2})}, \tag{S42}$$

where we have defined the functions

$$\begin{aligned} G(\omega_{\rm s1},\omega_{\rm s2},\pm\varOmega) &\doteq F_4(\omega_{\rm s1},\omega_{\rm s1},\omega_{\rm s2},\omega_{\rm s2}\mp\varOmega) - F_2(\omega_{\rm s1},\omega_{\rm s1})\,F_2(\omega_{\rm s2},\omega_{\rm s2}\mp\varOmega) \\ &= |\tilde{E}_{20}|^4\,\tilde{g}_2^2(\omega_{\rm s1}-\omega_{20})\,\tilde{g}_2(\omega_{\rm s2}-\omega_{20})\,\tilde{g}_2(\omega_{\rm s2}\mp\varOmega-\omega_{20})\,\tilde{G}(\omega_{\rm s2}-\omega_{\rm s1},\pm\varOmega) \end{aligned} \tag{S43}$$

and

$$\begin{aligned} \tilde{G}(\omega_{\rm R},\pm\varOmega) \doteq \int{\rm d}x\int{\rm d}x'\int{\rm d}y\int{\rm d}y'\,q_Q(x)\,q_Q(x')\,q_Q(y)\,q_Q(y') \\ \times[\tilde{F}_4(-x,-x',\omega_{\rm R}-y,\omega_{\rm R}\mp\varOmega-y') - \tilde{F}_2(-x,-x')\,\tilde{F}_2(-y,\mp\varOmega-y')]. \end{aligned} \tag{S44}$$

The correlation function $P_{I,S}(\omega_{\rm s1},\omega_{\rm s2},T)$ depends separately on $\omega_{\rm s1}$ and $\omega_{\rm s2}$ due to the presence of the Gaussian envelopes $\tilde{g}_2(\omega-\omega_{20})$. However, for sufficiently broadband pulses, the key features of $P_{I,S}(\omega_{\rm s1},\omega_{\rm s2},T)$ are determined by $\tilde{G}(\omega_{\rm s2}-\omega_{\rm s1},\pm\varOmega)$, which depends on $\omega_{\rm s1}$ and $\omega_{\rm s2}$ only via the Raman frequency

$$\omega_{\rm R} \doteq \omega_{\rm s2} - \omega_{\rm s1}. \tag{S45}$$

This is the equivalent of $\delta\omega$ in the main text. The function $\tilde{G}(\omega_{\rm R},\pm\varOmega)$ is responsible for the off-diagonal features observed in the experimental spectra of Fig. 1c in the main text. We calculate it numerically, including the effect of the finite resolution of the optical detector, and display it in the left panel of Fig. S6. The function exhibits a structure consisting of two main peaks,

$$\tilde{G}(\omega_{\rm R},\pm\varOmega) = \tilde{f}(\omega_{\rm R}) + \tilde{f}(\omega_{\rm R}\mp\varOmega), \tag{S46}$$

centered on $\omega_{\rm R} = 0$ and $\omega_{\rm R} = \pm\varOmega$, respectively. The shape of each peak, $\tilde{f}(\omega_{\rm R})$, is highlighted in the right panel of Fig. S6. It contains two narrow peaks, mostly determined by the correlation length R of the stochastic pulses used. We could check numerically that the function $q_Q(\omega)$, modeling the optical detector, influences the width of the two peaks and the depth of the central local minimum appearing in $\tilde{f}(\omega)$. However, this does not affect the central frequency of the peaks in $\tilde{G}(\omega_{\rm R},\pm\varOmega)$. Scanning the Raman frequency $\omega_{\rm R}$ allows one to identify the energy $\pm\varOmega$ of the phonons in the system.

The resulting function $P_{I,S}(\omega_{\rm s1},\omega_{\rm s2},T)$ is displayed in Fig. S7. Two peaks along $\omega_{\rm s2}-\omega_{\rm s1} = \pm\varOmega$ are clearly visible in the left panel, due to $\tilde{f}(\omega_{\rm R}\mp\varOmega)$ in $\tilde{G}(\omega_{\rm R},\pm\varOmega)$. In the right panel, we highlight the oscillations displayed by both peaks as a function of time delay. The oscillations along the two peaks take place at a frequency given by $\varOmega$ and have opposite phase, owing to the π shift between the initial coherences, A and A^*, generated by the pump pulse. The envelope $\tilde{g}(\omega_{\rm s}-\omega_{20})$ of the probe pulse determines the dependence of the standard deviations $\sigma_i(\omega_{\rm s})$ upon frequency. $\tilde{g}(\omega_{\rm s}-\omega_{20})$ and, thus, $\sigma_i(\omega_{\rm s})$ become very small far from the central frequency ω_{20}. The corresponding increase in the strength of the peaks is apparent in the left panel of Fig. S7.

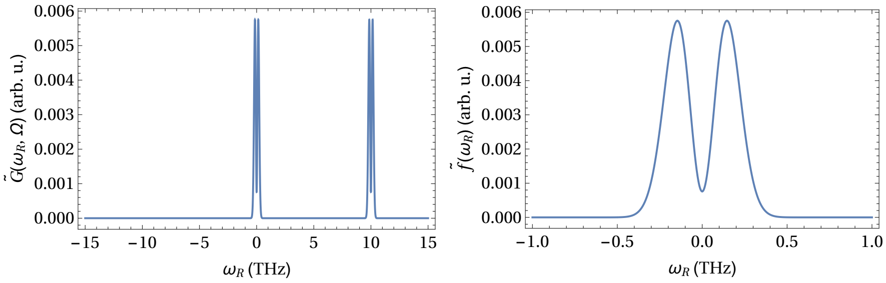

Figure S6. (left) $\tilde{G}(\omega_{\rm R}, \Omega)$ between the spectral function of the detected probe pulse evaluated at four different frequencies. (right) Peak $\tilde{f}(\omega_{\rm R})$ appearing $\tilde{G}(\omega_{\rm R}, \Omega)$.

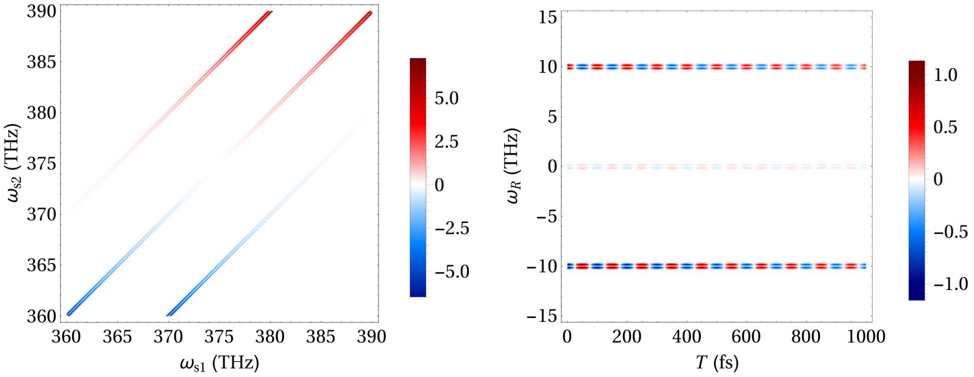

Figure S7. $P_{I,S}(\omega_{\rm s1}, \omega_{\rm s2}, T))$ for a fully modulated stochastic probe pulse. The cross-correlation function is displayed (left) as a function of the two frequencies $\omega_{\rm s1}$ and $\omega_{\rm s2}$ for $T = 500\,$fs, and (right) as a function of the Raman frequency $\omega_{\rm R}$ and time delay T for a mean frequency $(\omega_{\rm s1} + \omega_{\rm s2})/2 = 374\,$THz.

S5. AUTO- AND CROSS-CORRELATIONS FOR A STOCHASTIC PROBE WITH NOISE PARTIALLY DISTRIBUTED IN THE SPECTRAL BANDWIDTH

In this Supplementary Section, we consider a stochastic probe pulse with noise only partially distributed within the spectral width of the pulse, i.e.,

$$\tilde{\mathcal{E}}_{2,\rm sh}(\omega) = \tilde{E}_{20}\, \tilde{g}_2(\omega - \omega_{20}) \left[\theta(-(\omega - \omega_{\rm th})) + \theta(\omega - \omega_{\rm th})\, {\rm e}^{{\rm i}\varphi(\omega)}\right], \tag{S47}$$

where we have introduced the Heaviside step function $\theta(x)$ and the threshold frequency $\omega_{\rm th} = 370\,$THz, below which no spectral shaping is introduced. This corresponds to the experimental pulse of Fig. 1b in the main text. We assume $\langle \varphi^2 \rangle = (\pi/2)^2$ and model the finite resolution of the optical detector via the rectangular function in Eq. (S32), with $Q = 0.1\,$THz. We consider nonoverlapping pump and probe pulses, with the signal given by the ISRS spectrum $S_{\rm ISRS}(\omega_{\rm s}, T)$ of Eq. (S13), and study the dynamics of one single phonon of frequency $\Omega = 10\,$THz and lifetime $1/\gamma = 1.6\,$ps. For $\omega_{\rm s1}$ and $\omega_{\rm s2}$ both greater than the threshold frequency, the same auto- and cross-correlations are expected as in Sec. S4. However, when at least one of these two frequencies is lower than $\omega_{\rm th}$, a different behavior is observed. This is discussed in the following for the input-intensity autocorrelation and for the cross-correlation between input pulse and signal.

Figure S8 exhibits the autocorrelation function of the detected input intensity [Eq. (S37)]. For $\omega_{\rm s1} > \omega_{\rm th}$ and $\omega_{\rm s2} > \omega_{\rm th}$, the same peak along the main diagonal $\omega_{\rm s1} = \omega_{\rm s2}$ is found as in Fig. S5. For frequencies lower then $\omega_{\rm th}$, however, where the spectral phase is unshaped, the associated standard deviation $\sigma_{\rm in}(\omega_{\rm s})$ would vanish exactly in our model, and the associated Pearson coefficient $P(I_{\rm in}(\omega_{\rm s1}), I_{\rm in}(\omega_{\rm s2}))$ in Eq. (S37) would diverge. To avoid this divergence and obtain simulation results in agreement with the experiment, we assume here and in the following a small, finite standard deviation ϵ at frequencies $\omega < \omega_{\rm th}$ where the pulse does not undergo any modulation of the spectral phase. This simple assumption allows us to obtain results in agreement with the experimental analysis.

Figure S9 displays the cross-correlation $P_{I,S}(\omega_{\rm s1}, \omega_{\rm s2}, T)$ between the input intensity of the probe pulse and the ISRS signal. In order to understand the behavior of the cross-correlation function, we first observe that for $\omega_{\rm s1} < \omega_{\rm th}$, where the input intensity does not feature any added noise, no correlation can exist between $I_{\rm in}(\omega_{\rm s1})$ and the signal

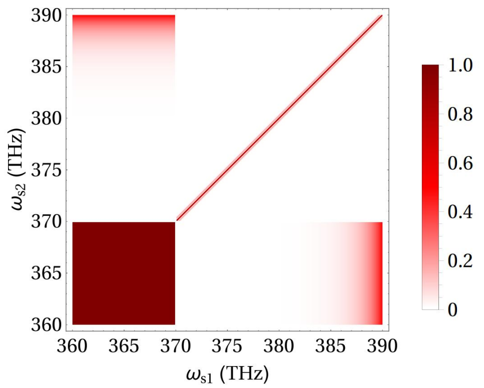

Figure S8. Pearson coefficient $P(I_{\rm in}(\omega_{\rm s1}), I_{\rm in}(\omega_{\rm s2}))$ of the autocorrelation of the detected input intensity for a partially modulated stochastic probe pulse.

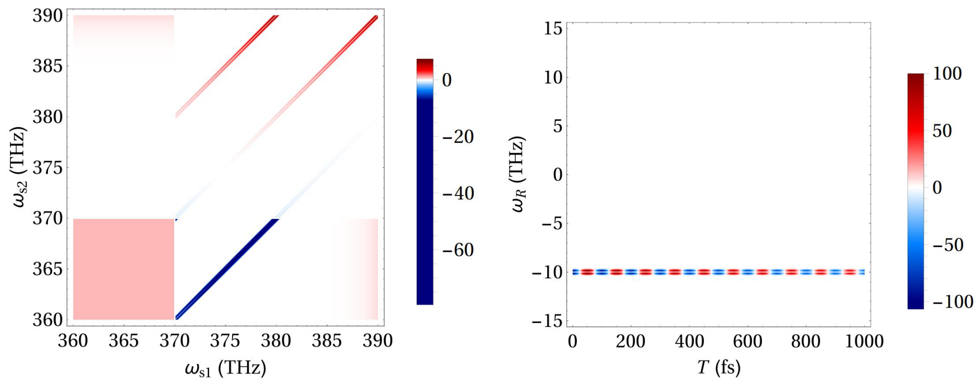

Figure S9. Contribution of the correlation function between the detected input intensity of the probe pulse and the ISRS signal to the Pearson coefficient $P(I_{\rm in}(\omega_{\rm s1}), I_{\rm out}(\omega_{\rm s2}, T))$ for a partially modulated stochastic probe pulse. The cross-correlation function is displayed (left) as a function of the two frequencies $\omega_{\rm s1}$ and $\omega_{\rm s2}$ for $T = 500\,{\rm fs}$, and (right) as a function of the Raman frequency $\omega_{\rm R}$ and time delay T for a mean frequency $(\omega_{\rm s1} + \omega_{\rm s2})/2 = 374\,{\rm THz}$. The colorbar in the left panel was chosen to highlight the weaker peaks appearing in the top-right quadrant.

$S_{\rm ISRS}(\omega_{\rm s2}, T)$, independent of the value of $\omega_{\rm s2}$. On the other hand, the signal is determined by the action of the stochastic probe pulse at frequencies $\omega_{\rm s2}$ and $\omega_{\rm s2} \pm \Omega$. For cross-correlations to appear, it is necessary that also these frequencies lie above the threshold frequency $\omega_{\rm th}$. In Fig. S9, three peaks can be distinguished. In contrast to Fig. S7, however, these peaks only appear when both contributing frequencies lie above the threshold frequency: (i) the central peak at $\omega_{\rm s1} = \omega_{\rm s2}$ is only present when both frequencies are larger than $\omega_{\rm th}$; (ii) the off-diagonal peak along $\omega_{\rm s1} = \omega_{\rm s2} - \Omega$ only appears if $\omega_{\rm s1} > \omega_{\rm th}$ and $\omega_{\rm s2} - \Omega > \omega_{\rm th}$; and (iii) the off-diagonal peak along $\omega_{\rm s1} = \omega_{\rm s2} + \Omega$ only appears for $\omega_{\rm s1} > \omega_{\rm th}$ and $\omega_{\rm s2} + \Omega > \omega_{\rm th}$. This creates a clear asymmetry in the cross-correlation function with respect to the Raman frequency $\omega_{\rm R}$, as visible in Fig. S9. Also in this case, we have assumed a small nonvanishing variance of the input and output intensities also for frequencies below $\omega_{\rm th}$, in order to avoid a vanishing denominator and divergences. This agrees with the experimental results, explaining the large strength of the peaks in the bottom-right quadrant of the left panel of Fig. S9 compared to the weaker peaks in the top-right quadrant.

S6. COMPARISON WITH TRADITIONAL PUMP-PROBE SPECTROSCOPY

We have performed mean-value based pump-probe measurements on the same alpha-quartz sample as a comparison to the covariance-based pump-probe spectroscopy technique. Table S1 summarizes the phonon dephasing times extracted for the three most prominent phonon modes within the pulse bandwidth used in these measurements. The mean-value and covariance-based measurements were performed one after the other, so experimental parameters could be kept consistent. The dephasing times are consistent within error margins between the two measurement techniques.

	3.9 THz	6.3 THz	13.9 THz
Covariance-based	2.67 ps	0.67 ps	1.22 ps
Mean-value	2.33 ps	0.45 ps	1.4 ps

Table S1. Phonon dephasing times measured using covariance based and mean-value based pump-probe techniques.

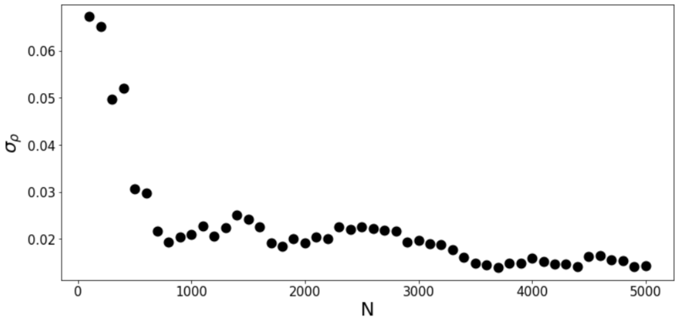

Figure S10. Standard deviation of a 15x15 pixel section of the covariance map for $\Delta T = -3\,ps$ in the quadrant where signal normally appears. This is a measure of the background due to spectrally uncorrelated noise in the detection system.

S7. UNCORRELATED BACKGROUND NOISE IN 2D CORRELATION MAPS

Spectrally uncorrelated noise introduced by the detection system (e.g. detector noise and electrical readout noise) produces a background contribution in the eventual 2D covariance maps that can obscure weak signals from the sample. However, this undesirable contribution to the measured spectra is relatively small compared to the real fluctuations in the probe pulse which contain the sample-induced correlations. In the correlation maps, the uncorrelated detection noise presents as a random signal nearly independent of ω_{OUT} and ω_{IN}, with positive and negative values distributed normally around zero. We can thus characterize the background noise level with a standard deviation of the values in areas of the covariance map where there is not signal present. This standard deviation of values decreases for increasing number of unique spectra acquired.

To quantify this effect in our measurement, we have taken the standard deviation (σ_ρ) of all points in a 15x15 pixel box within the lower right quadrant of the cross-correlation map (as indicated by the black dashed box in fig. 1c in the main text) for a single pump-probe delay. A pump-probe delay of -3 ps was chosen to ensure no signal was present. In Fig. S10 σ_ρ is plotted as a function of the number of unique spectra included in the calculation (N), showing a rapid decrease in σ_ρ below $N = 800$, followed by a slower decrease thereafter, tangentially approaching $\sigma_\rho = 0.01$. A thorough analysis of this noise is beyond the scope of this work, but it demonstrates that the signal (which has peak values of $\rho = \pm 0.1 - 0.2$) can be clearly resolved even with relatively low N.

A related question is how ρ can be connected to physically relevant quantities measured in traditional mean-value based experiments, such as $\Delta T/T$. Unfortunately there are intrinsic differences between the measurement techniques that hinder a direct connection. For example, ρ depends on two frequencies, whereas $\Delta T/T$ is measured at only a single frequency. Furthermore, the degree of correlation induced by a given signal depends on experimental parameters such as the amplitude and spectral width of the fluctuations introduced in the probe, and over what fraction of the probe bandwidth they are applied.

With that said, we have attempted to make a comparison between the results reported here with mean-value $\Delta T/T$ measurements performed on the same sample in similar conditions. In this case, we find that a correlation value of $\rho = 0.1$ is comparable to $\Delta T/T \approx 0.01$, but stress that this is not a universal relationship, and future work should be undertaken to understand in what circumstances ρ can be linked to $\Delta T/T$ or $\Delta R/R$ in a quantitative way.

[1] K. E. Dorfman, B. P. Fingerhut, and S. Mukamel, Time-resolved broadband Raman spectroscopies: A unified six-wave-mixing representation, J. Chem. Phys. **139**, 124113 (2013).
[2] C. A. Marx, U. Harbola, and S. Mukamel, Nonlinear optical spectroscopy of single, few, and many molecules: Nonequilibrium Green's function QED approach, Phys. Rev. A **77**, 022110 (2008).
[3] G. Fumero, G. Batignani, K. E. Dorfman, S. Mukamel, and T. Scopigno, On the Resolution Limit of Femtosecond Stimulated Raman Spectroscopy: Modelling Fifth-Order Signals with Overlapping Pulses, ChemPhysChem **16**, 3438 (2015).

[4] S. M. Cavaletto, D. Keefer, and S. Mukamel, High temporal and spectral resolution of stimulated x-ray Raman signals with stochastic free-electron-laser pulses, Phys. Rev. X **11**, 011029 (2021).
[5] N. Rohringer and R. Santra, X-ray nonlinear optical processes using a self-amplified spontaneous emission free-electron laser, Phys. Rev. A **76**, 033416 (2007).
[6] T. Pfeifer, Y. Jiang, S. Düsterer, R. Moshammer, and J. Ullrich, Partial-coherence method to model experimental free-electron laser pulse statistics, Opt. Lett. **35**, 3441 (2010).
[7] S. M. Cavaletto, C. Buth, Z. Harman, E. P. Kanter, S. H. Southworth, L. Young, and C. H. Keitel, Resonance fluorescence in ultrafast and intense x-ray free-electron-laser pulses, Phys. Rev. A **86**, 033402 (2012).
