# Supplementary figures and images for: Transient measurement of phononic states with covariance-based stochastic spectroscopy
Source: Light Sci Appl. 2022 Mar 1;11:44. doi: 10.1038/s41377-022-00727-6 (PMC8885707; doi:10.1038/s41377-022-00727-6)

Graphical abstract:


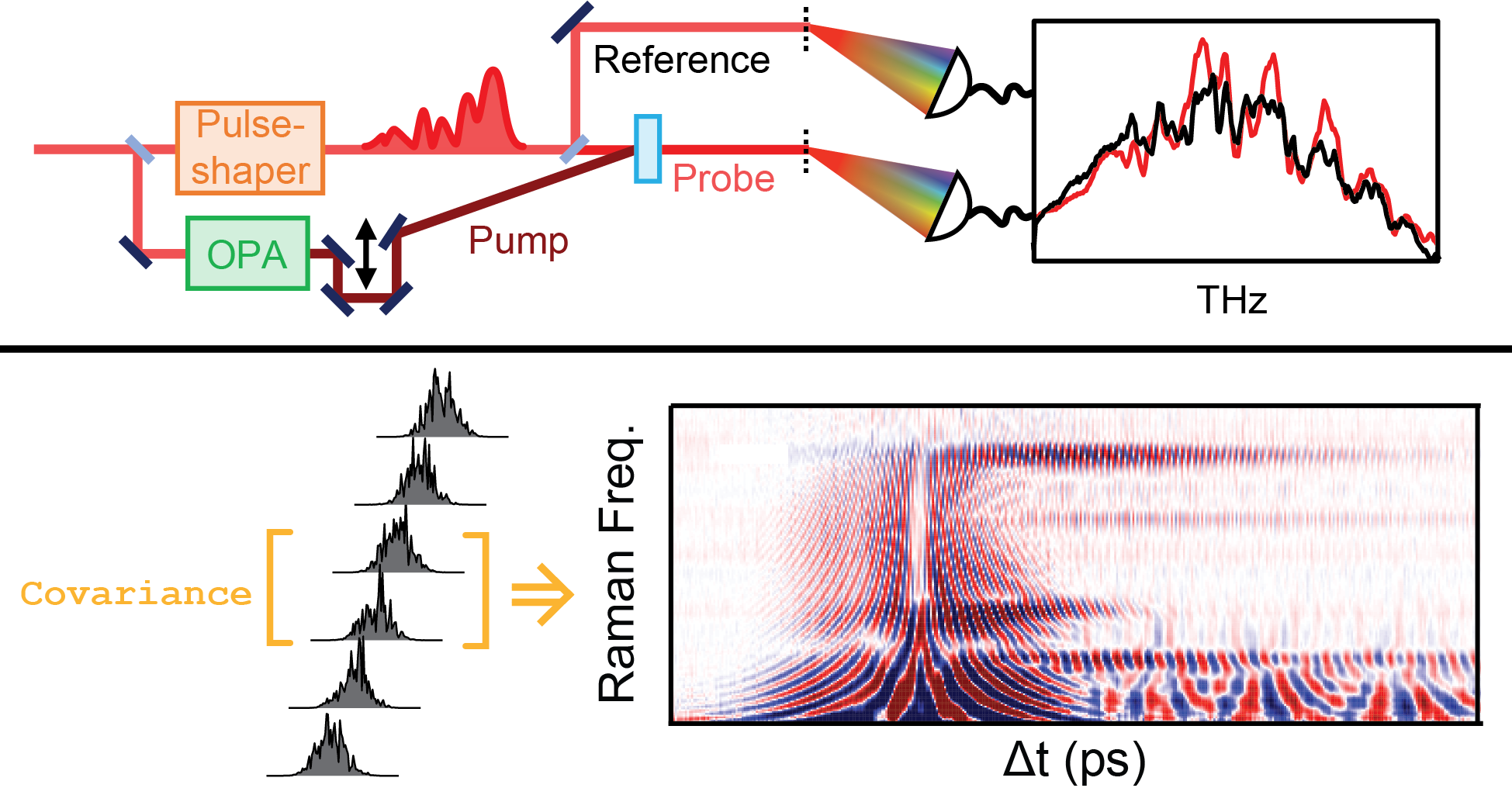

Supplement: Supplementary file 2 — Graphical Abstract [file 41377_2022_727_MOESM2_ESM.docx]
